# Reversible manipulation of the magnetic state in SrRuO_3_ through electric-field controlled proton evolution

**DOI:** 10.1038/s41467-019-13999-1

**Published:** 2020-01-10

**Authors:** Zhuolu Li, Shengchun Shen, Zijun Tian, Kyle Hwangbo, Meng Wang, Yujia Wang, F. Michael Bartram, Liqun He, Yingjie Lyu, Yongqi Dong, Gang Wan, Haobo Li, Nianpeng Lu, Jiadong Zang, Hua Zhou, Elke Arenholz, Qing He, Luyi Yang, Weidong Luo, Pu Yu

**Affiliations:** 1 State Key Laboratory of Low Dimensional Quantum Physics and Department of Physics, Tsinghua University, 100084 Beijing, China; 2 Key Laboratory of Artificial Structures and Quantum Control, School of Physics and Astronomy and Institute of Natural Sciences, Shanghai Jiao Tong University, 200240 Shanghai, China; 3 Department of Physics, University of Toronto, Toronto, ON M5S 1A7 Canada; 4 Advanced Photon Source, Argonne National Lab, Argonne, IL 60439 USA; 5 Materials Science Division, Argonne National Lab, Argonne, IL 60439 USA; 6 National Synchrotron Radiation Laboratory, University of Science and Technology of China, 230026 Hefei, Anhui China; 7 Beijing National Laboratory for Condensed Matter Physics, Institute of Physics, Chinese Academy of Science, 100190 Beijing, China; 8 Department of Physics and Astronomy, University of New Hampshire, Durham, NH 03824 USA; 9 Advanced Light Source, Lawrence Berkeley National Laboratory, Berkeley, CA 94720 USA; 10 Department of Physics, Durham University, Durham, DH13LE United Kingdom; 11 Frontier Science Center for Quantum Information, 100084 Beijing, China; 12 Collaborative Innovation Center of Advanced Microstructures, 210093 Nanjing, China; 13 RIKEN Center for Emergent Matter Science (CEMS), Wako, 351-198 Japan

**Keywords:** Electronic properties and materials, Ferromagnetism, Magnetic properties and materials

## Abstract

Ionic substitution forms an essential pathway to manipulate the structural phase, carrier density and crystalline symmetry of materials via ion-electron-lattice coupling, leading to a rich spectrum of electronic states in strongly correlated systems. Using the ferromagnetic metal SrRuO_3_ as a model system, we demonstrate an efficient and reversible control of both structural and electronic phase transformations through the electric-field controlled proton evolution with ionic liquid gating. The insertion of protons results in a large structural expansion and increased carrier density, leading to an exotic ferromagnetic to paramagnetic phase transition. Importantly, we reveal a novel protonated compound of HSrRuO_3_ with paramagnetic metallic as ground state. We observe a topological Hall effect at the boundary of the phase transition due to the proton concentration gradient across the film-depth. We envision that electric-field controlled protonation opens up a pathway to explore novel electronic states and material functionalities in protonated material systems.

## Abstract (web summary)

Ionic substitution is a useful way to manipulate structural, electronic, magnetic phase transitions in strongly correlated materials. Here, the authors report electric-field controlled protonation in SrRuO_3_, resulting in a large structural expansion and a ferromagnetic-to-paramagnetic phase transition.

## Introduction

Strong electron correlation and interplay between multiple degrees of freedom (charge, spin, orbital and lattice) give rise to a variety of fascinating electronic and magnetic phases in transition metal oxides, such as ferromagnetism, superconductivity, other charge (or spin) ordered states, etc^1–4^. Due to the extreme sensitivity of these systems to external stimuli, the ability to control versatile functionalities can achieve unique physical phenomena^5,6^. Among other oxides, SrRuO_3_ forms a fascinating material system for its interesting electronic and ferromagnetic properties^4^. Along the studies, the electric-field control of its magnetism, called magnetoelectric coupling^7–9^, has obtained particular research interests due to its associated intriguing physics and potential applications. Despite extensive explorations of dielectric^10,11^, ferroelectric^12,13^, and ionic liquid^14,15^ as gate layers during the electrostatic gating over the last two decades, the magnetism of SrRuO_3_ in these studies has only been altered slightly, and a deterministic electric-field control of its magnetic state has not been demonstrated yet.

Recently, there are also emerging research interests in SrRuO_3_ system on its exotic topological spin texture with the reports of the topological Hall effect (THE), which is attributed to the inequivalent interfaces in the studied ultra-thin films^11,13,16–20^. Furthermore, some recent results demonstrated nicely the electric-field controlled THE in ultrathin SrRuO_3_ films through the dielectric and ferroelectric modulations^11,13^, although the resultant effect remains subtle, reminiscent of its electric-field controlled magnetic state. Clearly, the distinct and rich magnetic transition correlated to the carrier density and inversion symmetry makes SrRuO_3_ a perfect model system to explore the electric-field controlled electronic and magnetic phase diagram, which might trigger a wide range of device applications.

Here, we demonstrate an efficient and reversible tunability of both the structural and electronic phase transformations within SrRuO_3_ thick film through electrically controlled protonation during the ionic liquid gating. With increasing protonation concentration in this compound, the ferromagnetism was gradually suppressed, and eventually we discover a novel protonated compound of HSrRuO_3_, which shows an exotic paramagnetic metallic ground state. In addition, a pronounced tunable THE is observed near the boundary of the phase transformation, which suggests an effective strategy to design THE in this compound.

## Results

### Gate tunable structural transformation via proton evolution

**Fig. 1:**
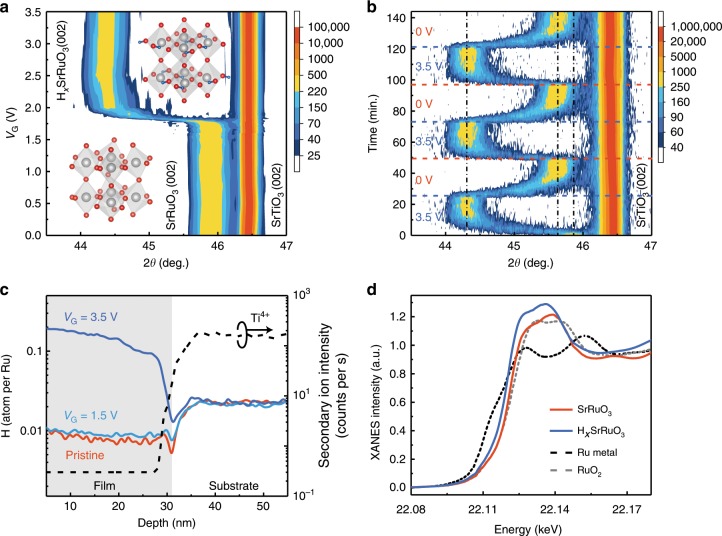
Electrically controlled proton evolution in SrRuO_3_ films. **a** In-situ XRD *θ-2θ* scans around the SrRuO_3_ (002) peak as a function of *V*_G_. The new phase is denoted as H_*x*_SrRuO_3_. The insets show calculated crystal structures of SrRuO_3_ and HSrRuO_3_, respectively, where the blue balls represent hydrogen atoms. **b** In-situ XRD *θ-2θ* scans around the SrRuO_3_ (002) peak as *V*_G_ cycled between 3.5 V and 0 V, indicating the reversibility of the structural phase transformation. The black dotted lines indicate the peak positions, and the blue and red dash lines denote the change of *V*_G_. **c** Hydrogen distribution profiles within both pristine SrRuO_3_ film (red solid line) and gated (with *V*_G_ = 3.5 V and 1.5 V) SrRuO_3_ films, as measured by ex-situ SIMS. The Ti^4+^ signature was used as a marker to define the interface between film and substrate. **d** In-situ XANES spectra at Ru K-edge for pristine SrRuO_3_ (orange solid line) and protonated H_*x*_SrRuO_3_ (blue solid line) at *V*_G_ = 3.5 V. The XANES spectra at Ru K-edge for Ru metal (black dash line) and RuO_2_ (gray dash line) are shown as references.

Our experiments were performed on high quality epitaxial SrRuO_3_ films grown on SrTiO_3_ (001) substrates by pulsed laser deposition (see Methods section). To explore the tunability of electrically controlled protonation, we first performed an in-situ X-ray diffraction (XRD) measurements during the ILG, in which a positive voltage would drive the positively charged protons into the film. Figure 1a shows the gate voltage (*V*_G_) dependent *θ–*2*θ* scans around the SrRuO_3_ (002) peak, in which the SrRuO_3_ (002) peak shows no obvious shift with *V*_G_ up to ~1.8 V, while a further increase of *V*_G_ leads to a clear shift of the peak position to a lower angle (from 45.90° to 44.35°). This result suggests a large out-of-plane lattice expansion up to 3.3% for the SrRuO_3_ film, which is comparable with our recent result of pronation induced phase transformation from SrCoO_2.5_ to HSrCoO_2.5_^21^. It is interesting to note that when the gate voltage is removed, the diffraction peak returns nearly back to the original position with a slight offset, and afterwards the phase transformation can be reversibly and reproducibly controlled with the application of positive *V*_G_ (3.5 V) and zero voltage (Fig. 1b). Importantly, the in-plane lattice constants and crystalline quality of the film remain unchanged throughout ILG as evinced by the reciprocal space mapping, rocking curves, and reflectivity measurements (Supplementary Fig. 1). Notably, the structural transformation possesses a threshold gating voltage, as well as a clear voltage dependence with higher voltage corresponding to shorter transition time (Supplementary Fig. 2), which is consistent with the diffusion model suggested by previous studies^21–23^.

As our film thickness is much larger than the screening length associated with electrostatic gating, we can readily single out the ionic (H^+^ or O^2−^) evolution as the dominant mechanism for the observed structural phase transformations. To identify the type of ion responsible for the phase transformation, ex-situ secondary-ion mass spectrometry (SIMS) was performed, as shown in Fig. 1c and Supplementary Fig. 3a. The result shows considerable numbers of protons distributed in the SrRuO_3_ film associated with a structural transformation after being gated at *V*_G_ = 3.5 V, but not in the pristine sample or in the film gated at 1.5 V. On the other hand, the ^18^O isotopic calibration measurements^24^ suggest that the oxygen ion evolution is negligible for the gated samples (Supplementary Fig. 3b). Therefore, we can conclude that the ILG induced structural transformation is strongly associated with the protonation evolution. Finally, the reason why protons leave the sample at zero voltage should be attributed to the phase instability of H_*x*_SrRuO_3_. Although some protonated phases (e.g., H_*x*_SrCoO_2.5_) are nonvolatile and can remain stable in air even with the gating voltage turned off^21^, other protonated materials (e.g., H_*x*_WO_3_) do possess the volatile nature with a reversible phase transformation back into almost pristine state when the gate voltage is removed^22^. In the latter case, the detected hydrogen signal is attributed to the residual portion of protons in the film.

Crucially, the presence of positively charged protons are known as electron donor within the materials^21,22^. To trace the associated valence state evolution in Ru, we performed in-situ hard X-ray absorption experiments near Ru *K*-edges for both pristine and protonated (with a gate voltage of 3.5 V) samples, as shown in Fig. 1d, along with two referenced compounds (RuO_2_ and Ru metal)^25^. Clearly, a significant energy shift towards lower energy region was observed with respect to that of the pristine state, suggesting the reduction of the Ru valence state from +4 to +3 due to the electron doping associated with protonation.

### Reversible control of magnetism through proton evolution

**Fig. 2:**
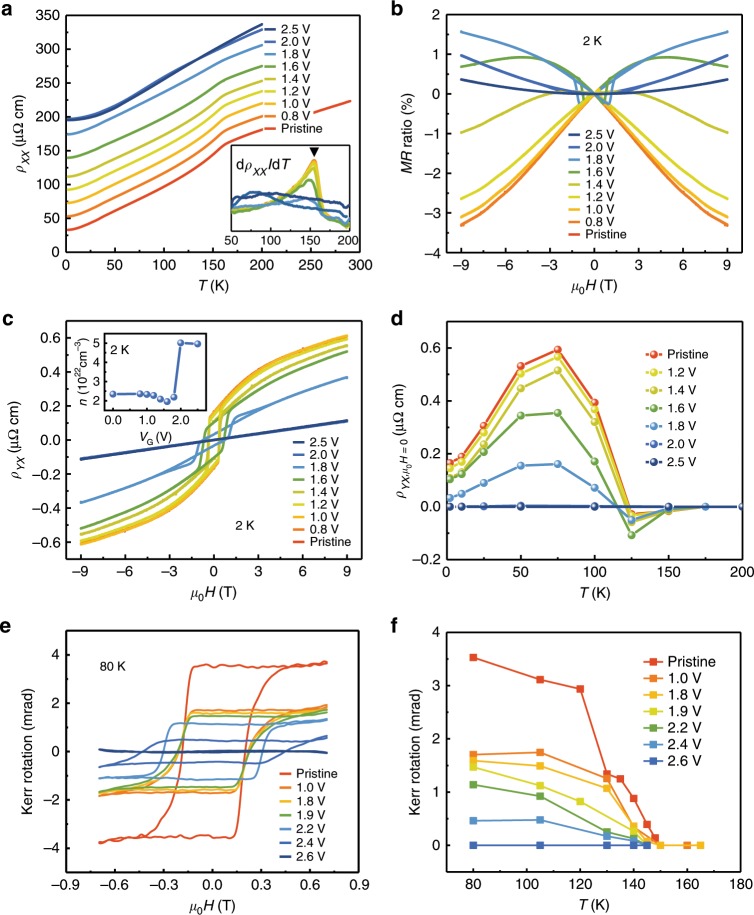
Magnetic evolution via electrically induced protonation. **a** Temperature dependent longitudinal resistivity $\rho _{XX}$ at different *V*_G_. The inset shows the corresponding differentiate resistivity d $\rho _{XX}/{\mathrm{d}}T$ at different *V*_G_. A vertical offset of 20 μΩ cm is applied for each curve for clarity. **b** Magnetic field dependent magnetoresistance (MR) measured at 2 K with different *V*_G_. **c** Magnetic field dependent Hall resistivity measured at 2 K with different *V*_G_. The inset shows the *V*_G_ dependence of carrier density at 2 K. **d** Temperature dependent anomalous Hall resistivity obtained at *μ*_0_*H* = 0 T with different *V*_G_. **e** Kerr rotation vs. magnetic field results measured at 80 K with different *V*_G_. **f** Kerr rotation as a function of temperature obtained at *μ*_0_*H* = 0 T with different *V*_G_. The slightly varied threshold gate voltages among transport, MOKE and XRD measurements are attributed to the different device configurations.

Since the phase transformation can be gradually controlled during ILG, the current study provides a unique opportunity to investigate the evolution of electronic state in SrRuO_3_ through protonation. Figure 2a shows the temperature dependent resistivity *ρ*_*XX*_(*T*) for SrRuO_3_ with different *V*_G_ during ILG, in which the thin film remains metallic throughout the gating. However, a careful analysis reveals that the kink feature, which can be observed at ~160 K (Curie temperature *T*_C_) for the pristine sample, gradually smooths out and eventually disappears (inset of Fig. 2a). These results suggest a possible suppression of ferromagnetism during ILG, as the kink feature is a typical characteristic for ferromagnetism in SrRuO_3_. This magnetic transition can also be observed in the magnetoresistance (MR = *ρ*_*XX*_(*H*)/*ρ*_*XX*_(0)−1) measurements, as shown in Fig. 2b. As *V*_G_ increases, the typically negative butterfly-like MR gradually decreases, and more interestingly, with the gating voltage of 2.5 V, we observed a positive parabolic MR, representing a conventional paramagnetic metallic state.

To clearly investigate the evolution of the ferromagnetic state in SrRuO_3_ under ILG, we measured the magnetic-field dependent Hall resistivity at different *V*_G_. The pristine SrRuO_3_ film exhibits a well-defined hysteresis loop attributed to the anomalous Hall effect (AHE) associated with the ferromagnetic state. As *V*_G_ increases, the hysteresis loop (at 2 K) is gradually suppressed and eventually turns into a linear response with *V*_G_ of 2.5 V, as shown in Fig. 2c. Figure 2d summarizes the AHE resistivity (extracted at *μ*_0_*H* = 0 T) at different temperatures under various *V*_G_, in which the anomalous Hall resistivity gradually decreases and eventually disappears with the increase of *V*_G_. Furthermore, the electron carrier density increases by about 2.61 × 10^22^ cm^−3^ from pristine to the 2.5 V gated sample (insert in Fig. 2c), which is consistent with the change of Ru valence state from +4 to +3 (corresponding to 1.65 × 10^22^ cm^−3^), and such a significant increase of carrier density further supports the scenario that the intercalated hydrogen serves as an effective electron donor into SrRuO_3_ system. It is interesting to note that the diffusion process would also be strongly correlated with the gating temperature, leading to a dramatically different magnetic state for the sample gated at different temperature (Supplementary Fig. 4).

The magnetic evolution during ILG was also studied with the in-situ magneto-optic Kerr effect (MOKE) measurements, which measures the ac inter-band Hall conductivity and has the same origin as the intrinsic AHE (i.e., the anomalous velocity due to the Berry curvature in momentum space^26^). Similar to the Hall measurements, as *V*_G_ increases the square-like MOKE hysteresis loop is gradually suppressed and eventually disappears (Fig. 2e, f), indicating that the ferromagnetism is indeed weakened by the ILG induced protonation. Furthermore, the element-specific X-ray magnetic circular dichroism (XMCD) measurements at the Ru *L*_3,2_ edges clearly show the suppression of ferromagnetism in Ru ions in the protonated sample (Supplementary Fig. 5). Undoubtedly, all these experimental observations provide strong evidences that the protonated H_*x*_SrRuO_3_ sample undergoes an exotic ferromagnetic to paramagnetic phase transition with the protonation induced electron modulation. More importantly, we revealed a novel protonated compound of HSrRuO_3_ with paramagnetic metallic as ground state. Similar to the structural transformation, the modulation of the ferromagnetic state is also reversible when cycling *V*_G_ (Supplementary Fig. 6). The slight suppression of AHE signal (as well as the magnetization) after removing the gating voltage as compared to the pristine samples (Supplementary Fig. 6 and Supplementary Fig. 7) should be attributed to the residual protons previously observed in the structural modulation and SIMS measurements (Supplementary Fig. 1b, c).

**Fig. 3:**
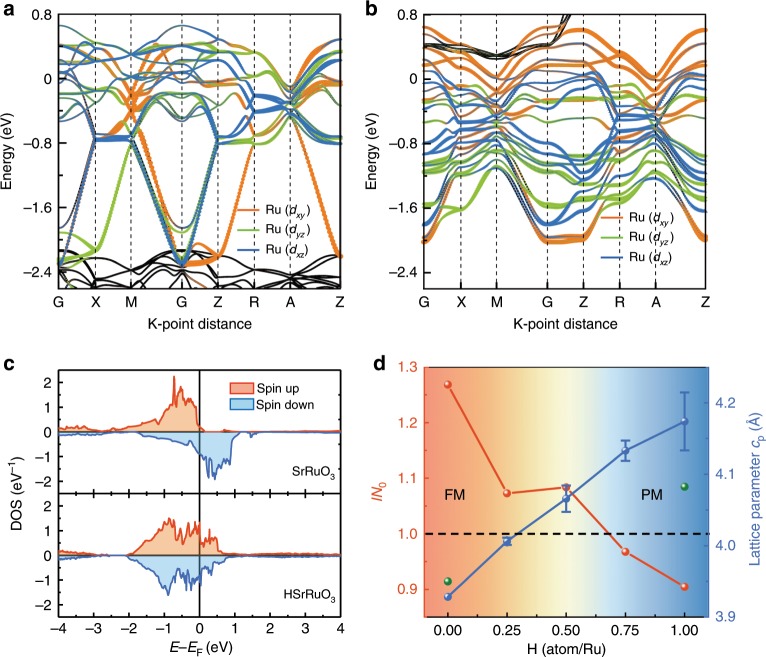
Mechanism for the protonation induced magnetic phase transition. **a**, **b**, Calculated electronic band structures for (**a**) pristine SrRuO_3_ and (**b**) HSrRuO_3_ with nonmagnetic General Gradient Approximation (GGA) calculations. **c** Spin-resolved density of states for pristine SrRuO_3_ and protonated HSrRuO_3_ calculated by GGA. **d** Calculated Stoner criterion parameter *IN*_0_ and *c*-axis lattice parameter as a function of hydrogen concentration. The green points are the experimental lattice parameters (doubled of the pseudo-cubic lattice constant) obtained from the in-situ XRD measurements. Following the Stoner criterion, when *IN*_0_ becomes smaller than 1 with the increase of hydrogen concentration, the ferromagnetic (FM) SrRuO_3_ transits into a paramagnetic (PM) metal. The error bars are calculated from different possible H_*x*_SrRuO_3_ crystalline structures.

To shed more light on the protonation induced magnetic transition, we carried out first-principles calculations (see Methods section). The optimized crystalline structure for HSrRuO_3_ is shown in the inset of Fig. 1a, in which the proton is bonded with the equatorial oxygen of Ru octahedral as the ground state, while its bonding with apical (or mixed equatorial and apical) oxygen would lead to higher system energy (Supplementary Fig. 8). Figures 3a, b present the calculated non-spin-polarized band structures for pristine and protonated HSrRuO_3_ samples, respectively. Clearly, the proton intercalation leads to a dramatically modified density of states (DOS) due to the significant splitting of the degenerated Ru *t*_2g_ bands and shift of spectra weight toward lower energy. As shown in Fig. 3c, although the spin-resolved DOS shows significant splitting of majority (down) and minority (up) bands in pristine SrRuO_3_, the corresponding DOS in protonated HSrRuO_3_ shows a nearly equivalent spectral weight, indicating the absence of ferromagnetism in the latter. It has been established that the metallic ferromagnetism of SrRuO_3_ can be described within the framework of Stoner model^4,27^, in which the ferromagnetic ground state is favored when *IN*_0_ > 1, where *I* and *N*_0_ are the so-called Stoner factor and nonmagnetic DOS per spin at the *E*_F_, respectively. Accordingly, we calculated crystalline structures, as well as Stoner factors (and then *IN*_0_ value) for a series of protonated phases with different proton concentrations, as summarized in Fig. 3d. The results show that with increasing proton concentration, the lattice results in a dramatic expansion, being consistent with the XRD results, and the value of *IN*_0_ gradually decreases. According to the Stoner criterion, a non-magnetic (or paramagnetic) ground state would be favored for the case with *IN*_0_ < 1, therefore this theoretical calculation nicely explains our experimental observations of protonation induced ferromagnetic to paramagnetic transition in the SrRuO_3_ film.

As the protonation process can result in both electron doping and lattice expansion, we further calculated the Stoner parameters for cases with charge modulation and structural expansion independently involved, and the corresponding DOS results are summarized in Supplementary Fig. 9. In the former case, the Stoner parameter is 1.06 (1.09) for adding 0.5 (1.0) electron per Ru, indicating a rather stable ferromagnetic state. This calculation can also explain the reason why the ferromagnetism is so robust even for ultrathin SrRuO_3_ during the electrostatic gating^10–12,14,15,28^. In the latter case, the Stoner parameter equals to 1.28 when only the lattice expansion (5.4%, as suggested by the theoretical computation in HSrRuO_3_) is considered, suggesting ferromagnetic phase as the ground state. This is indeed reasonable considering the fact that BaRuO_3_ is also ferromagnetic despite of large chemical expansion as compared with SrRuO_3_^29^. With these extended calculations, it is clear that neither the charge modulation nor the structural expansion alone could lead to the observed ferromagnetic to paramagnetic transition, which clearly highlights the unique role of protonation in SrRuO_3_ system. More importantly, the protonated SrRuO_3_ forms a new material paradigm with a paramagnetic ground state, which further suggests protonation as an effective pathway to engineer Ru based oxide systems (e.g., Sr_2_RuO_4_ and CaRuO_3_) through protonation induced electron doing, as well as structural deformation.

### THE induced by proton concentration gradient

**Fig. 4:**
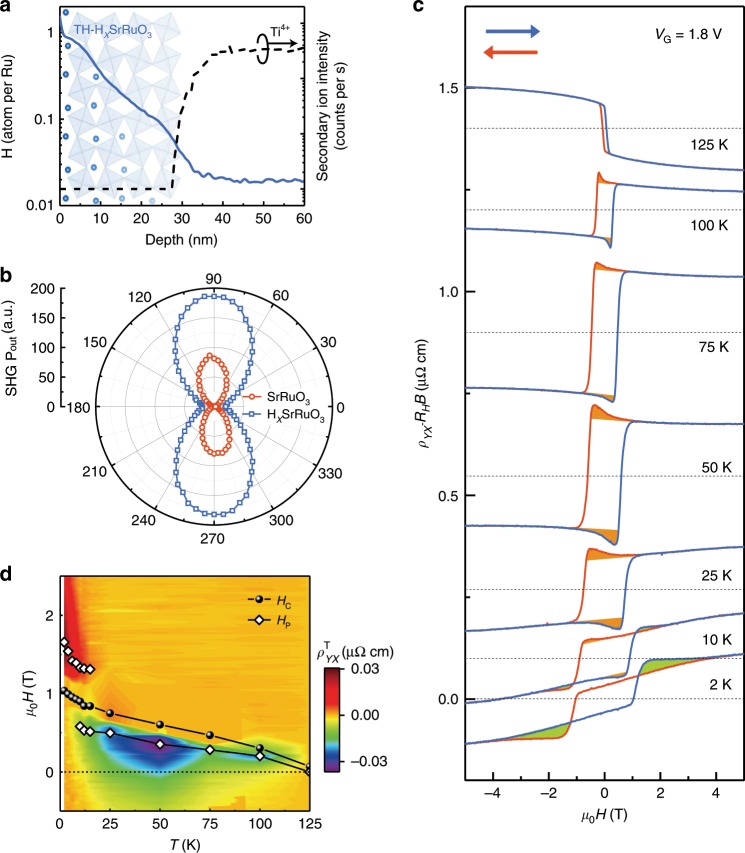
Emergence of topological Hall effect in H_*x*_SrRuO_3_. **a** Ex-situ measured proton distribution profile in a sample (~32 nm) with topological Hall effect. **b** P-polarized SHG intensity as a function of the polarization direction of the incident light (0 corresponds to s-polarization) for both pristine SrRuO_3_ and gated H_*x*_SrRuO_3_ films. The weaker SHG in the pristine film is due to surface contributions, while the enhanced SHG intensity of the H_x_SrRuO_3_ state suggests the breaking of inversion symmetry in bulk. **c** Magnetic field dependent Hall resistivity for H_*x*_SrRuO_3_ gated with *V*_G_ = 1.8 V at different temperatures. The blue and red arrows denote the field sweeping direction. Ordinary Hall term is subtracted through the linear fitting of *R*_H_*B* at higher magnetic fields. An offset is applied per curve for clarity, while the dotted lines denote the center of the hysteresis loops. The estimated topological Hall resistivity with different signs is marked with different colors. **d** Color map of estimated topological hall resistivity $\left( {\rho _{YX}^{\mathrm{T}}} \right)$ and characteristic fields (*H*_C_ and *H*_P_) obtained at H_*x*_SrRuO_3_ gated with *V*_G_ = 1.8 V. *H*_C_ (black filled symbol) represents the coercive field and the *H*_P_ (white open diamond) denotes the field where the topological Hall resistivity reaches its maximum.

Knowing the fact that the structural transformation is dominated through the proton diffusion process (Supplementary Fig. 2 and Supplementary Fig. 4), we further developed a novel strategy to manipulate the structural symmetry of SrRuO_3_ during ILG. To clearly capture the whole picture of the ILG induced phase transformation, a detailed in-situ XRD study was carried out for a thicker (~90 nm) sample during the ILG. With fine tunings of the gating voltage, we observe a dramatic broadening of XRD diffraction (002) peak at certain voltage (Supplementary Fig. 10a, b), suggesting the formation of inhomogeneous protonation along the film normal at intermediate state with suppressed ferromagnetism. Accordingly, we observed a considerable proton concentration gradient in an ex-situ H_*x*_SrRuO_3_ sample (Fig. 4a) formed at the boundary of magnetic transition. These results indicate a straightforward strategy to break the inversion symmetry in the current system. Indeed, an increased second harmonic generation (SHG) signal was also detected in the H_*x*_SrRuO_3_ state as compared to the pristine film (Fig. 4b and Supplementary Fig. 10c), which can be explained by the fact that the broken inversion symmetry allows the bulk, rather than just the surface, to contribute to the SHG signal. It is important to point out that our extensive calculations reveal that the ground state of SrRuO_3_ remains nonpolar through protonation due to its metallic nature (Supplementary Fig. 11), therefore, the ILG induced protonation provides a novel pathway to control the inversion symmetry within SrRuO_3_, in which the proton concentration gradient leads to a broken inversion symmetry through a built-in polarization field.

It has been established that the SrRuO_3_ itself has a rather large spin-orbit coupling^4^, and recent studies demonstrate the breaking of inversion symmetry by inequivalent interfaces in ultrathin SrRuO_3_ system can result in large Dzyaloshinskii-Moriya (DM) interaction with the emergent THE^11,13,16–18,20,30^. Then one would immediately realize that our current system provides a suitable condition to manipulate the THE effect.

Interestingly, a close view of Fig. 2c reveals indeed that a distinct hump feature superposing on the AHE loop at *V*_G_ 1.8 V (also see Fig. 4c). The fact that the in-situ Kerr signal exhibits only the conventional magnetization loops (Fig. 2e) as compared with the Hall measurements showing additional hump feature at the same temperature (Supplementary Fig. 11) suggests that this hump feature has a strong frequency dependence as observed by our recent studies^31^. We attribute the hump feature to the THE, as similar feature was widely observed in bulk MnSi^32,33^, EuO thin films^34^ and ultrathin SrRuO_3_ film^11,13,16–18,20,30^ as a hallmark of THE.

To quantitatively evaluate the THE, we estimated the topological Hall resistivity $\rho _{YX}^T$ (as shown in Fig. 4c) by subtracting the AHE signal through linear fitting of the high field data. With this, we can construct a phase diagram for the topological Hall term $\rho _{YX}^T$ in the *T*-*H* plane (Fig. 4d), showing that the THE clearly exists in a wide range of the *T*-*H* plane. Moreover, although the sign of the AHE component remains unchanged up to 100 K, the corresponding THE component changes sign with the increase of temperature. In particular, both positive and negative THE components can be identified at certain temperatures (e.g., 10 K) In addition, we confirm that the THE is driven by the magnetization reversal process due to the fact that the peak position of THE (*H*_P_) scales nicely with the coercive field (*H*_C_). We note that in previous studies of ultra-thin SrRuO_3_ film systems, the THE emerges as the consequence of the enhanced DM interaction, as well as reduced ferromagnetism due to the interface effect^11,16,17^. Similarly, in our system, the ILG induced large proton compositional gradient at the boundary of the ferromagnetic to paramagnetic phase transition would lead to an enhanced DM interaction as well as reduced ferromagnetism, and both favor the emergence of THE.

As it is demonstrated that the THE emerges during the ILG induced magnetic transition, one could expect that similar effect would also occur during the magnetic transition from paramagnetic to ferromagnetic phase. To confirm that, we performed in-situ AHE measurements during the proton out-diffusion process from a fully gated HSrRuO_3_ sample with the gating voltage turned off (Supplementary Fig. 12). The result clearly demonstrates that the AHE signal recovers gradually toward its pristine state as a function of dwell time, and more importantly a distinct THE emerges at the phase boundary.

## Discussion

In addition, we want to point out that some recent works attributed the observed hump AHE features in ultra-thin SrRuO_3_ films as a trivial mixture of AHE hysteresis loops with positive and negative components due to co-existence of multiple domains or berry curvatures^35–37^, and similar two-component AHE was also reported in magnetically doped topological insulator^38^. In these cases, the hump feature (or “THE”) would appear only in a narrow temperature region with switching AHE polarity, which is indeed observed in most works with ultra-thin SrRuO_3_ films. This issue makes the underlying mechanism for the observed “THE” in ultra-thin SrRuO_3_ films a hotly debated topic recently.

However, the THE observed in our study is independent of the sign of AHE and persists through the whole temperature region below *T*_C_, which could not be explained by the trivial mixed AHE model. With these differences, we are confident that the current result represents a novel strategy to engineer the THE in SrRuO_3_. It is interesting to note that using magnetic force microscope (MFM), some studies observe the nanoscale skyrmion in the ultra-thin SrRuO_3_ heterostructures^13,16,39^, and therefore attributed the observed THE to the formation of skyrmion. We speculate that this also forms a plausible explanation for our result since our sample holds all essential ingredients required to host Skyrmion: broken inversion symmetry, strong spin-orbital coupling and reduced magnetization. However, since the protonated sample is covered entirely by the ionic liquid and the phase transformation is volatile, we are not able to directly measure its magnetic domain structure through MFM or Lorentz transmission electron microscope^40^ techniques. Therefore, we are not able to make a conclusive assignment of the mechanism for the observed THE in protonated SrRuO_3_ at present, and further study would be highly demanded to clarify this issue.

To summarize, our work demonstrates an effective control of magnetism and THE in SrRuO_3_ system through the electric-field induced protonation. The discovered chemical stabled HSrRuO_3_ suggests a new strategy to engineer the structural, electrical and magnetic state in complex oxides. We envision that our study opens up possibility for the exploring of exotic physics and potential applications in wide range of protonated material systems.

## Methods

### Film growth and XRD measurements

Epitaxial SrRuO_3_ films were grown on (001) SrTiO_3_ and (001) LaAlO_3_ substrates (only for in-situ XANES measurements) by pulsed laser deposition (KrF, λ = 248 nm). All films were deposited at identical conditions with a substrate temperature of 700 ℃ and an oxygen partial pressure of 100 mTorr. The energy density of laser was fixed at 2 J/cm^2^ with growth rate of 1.7 nm/min. To minimize the oxygen vacancy concentration, samples were post-annealed at growth temperature for 15 min and then cooled down to room temperature at a cooling rate of 10 ℃ per minute in an atmosphere of oxygen pressure. For in-situ X-ray diffraction (XRD) measurements, Au electrodes were sputtered at edges of films, and a slice of Pt was employed as the gated electrode. Both sample and Pt were placed in a quartz bowl, and then the whole gating device was put on the XRD sample stage. The covered ionic liquid (IL) was carefully controlled so as to get strong enough diffracted X-ray signal. The in-situ *θ-*2*θ* and reciprocal space mapping (RSM) measurements were performed with a high-resolution diffractometer (Smartlab, Rigaku) using monochromatic Cu Kα1 radiation (λ = 1.5406 Å) at room temperature. The *V*_G_ dependent *θ-*2*θ* scans were measured with the same time interval for each curve.

### Electrical transport and magnetic measurements

The transport measurements were performed in a PPMS setup (Quantum Design DynaCool system, 9T) equipped with lock-in amplifiers (Model LI 5640, NF Corporation). Hall-bar structures (220 μm × 60 μm) were fabricated by standard lithography and Au/Ti was sputtered as electrodes. The device was placed in a quartz bowl covered entirely with IL and a slice of Pt was used as the gate electrode. To exclude the offsets for Hall resistivity and longitudinal resistivity due to misalignment of contacts, the Hall resistivity $\rho _{YX}$ and longitudinal resistivity $\rho _{XX}$ were calculated from the raw data as:

1 $$\rho _{YX} = - t[V_H\left( { + H \to - H} \right) - V_H\left( { - H \to + H} \right)]/2I$$

2 $$\rho _{XX} = Wt[V_R\left( { + H \to - H} \right) + V_R\left( { - H \to + H} \right)]/2IL$$

where *t, W* (60 μm)*, L* (220 μm) are the film thickness, width and length of the device, respectively, and *I* is the excitation current. *V*_G_ was applied between the gate electrode (Pt) and the SRO film. For each *V*_G_, the measured conditions were the same. The *V*_G_ was changed at 290 K and then dwelled for 25 min for each cycle. Magnetic characterizations, including the temperature-dependent magnetization (*M–T*) and magnetic hysteresis loops (*M–H*), were carried out with a 7T SQUID magnetometer (Quantum Design). Both electrical and magnetic measurements were done within vacuum cryostats.

### In-situ MOKE measurements

The magnetic-optical Kerr effect (MOKE) measurements were carried out using a narrowband continuous-wave diode laser at 895 nm wavelength. The sample was mounted on the cold finger of a small optical cryostat (3–300 K). One edge of the sample was painted with the silver conductive adhesive as the bottom electrode, while platinum wire surrounding the sample was used as gate electrode. The sample was then covered by a droplet of ionic liquid and then further covered by a coverslip to obtain flat surface. Magnetic field (up to 0.7T) generated using an external electromagnet was applied perpendicular to the sample plane (Faraday geometry). The light (250 μW) was linearly polarized and focused weakly into a 50 μm spot on the sample surface at normal incidence. The out-of-plane magnetization of the sample was then detected by measuring the optical Kerr rotation (*θ*_K_) of the reflected light. The light intensity was modulated with a mechanical chopper at 1.2 kHz to facilitate a lock-in detection. The Kerr rotation signals as a function of the applied magnetic field were measured with a standard optical bridge arrangement using balanced photodiodes.

### Second harmonic generation (SHG) measurements

To measure the SHG signal, a Ti: Sapphire pulsed laser (800 nm wavelength, 200 fs pulse duration, 76 MHz repetition rate, average power 40 mW) was linearly polarized and then focused onto the sample at a 45° incident angle using a 0.35 NA microscope objective. The reflected beam was collimated and passed through a linear polarizer and two 450 nm short pass filters to remove all laser light at 800 nm. The second harmonic (400 nm) light was then detected using a photomultiplier tube connected to a current preamplifier. The signal was modulated at approximately 3 kHz using an optical chopper and detected with a lock-in amplifier to improve the signal to noise ratio. For each setting of the final linear polarizer, the SHG power was measured as a function of the initial polarization angle of the laser. The sample was also mounted on a manual rotation stage to allow different orientations of the sample with respect to the plane of incidence to be measured.

### ^18^O isotopic calibration measurements

To trace oxygen ion evolution during the ILG, we carried out ^18^O isotopic calibration measurements on SrRuO_3_ samples. The ILG setup was kept in a closed chamber which was filled by ^18^O_2_ gas. Then we gated the thin film with V_G _= 3.5 V for an hour which was enough for the structural phase transformation. After the ILG, we removed the gate voltage and kept the sample in ^18^O_2_ chamber for an hour to let it fully relaxed toward the pristine state. Since the existence of oxygen exchange between the ionic liquid and the ^18^O_2_ atmosphere, if any oxygen ion evolution is induced during the structural phase transformation, we would be able to detect notable ^18^O residual within the gated sample. However, no obvious difference for the ^18^O signals between the sample gated in ^18^O_2_ atmosphere and the pristine sample clearly suggests that the oxygen ion evolution is negligible during the ILG in SrRuO_3_.

### Compositional analysis through the phase transformation

The secondary ion mass spectroscopy (SIMS) measurements for H, ^18^O, and deuterium (D or ^2^H) were carried out using a TOF-SIMS 5–10 instrument (IONTOF GmbH). The sputtering area was 250 μm × 250 μm and the detecting area was kept as 50 μm × 50 μm to avoid the disturbance from crater edges. The position of heterointerface was indexed by measuring Ti element from SrTiO_3_ substrate. All samples were measured at the same condition. The concentration of H was estimated by profiling proton-implanted SiO_2_ with known hydrogen dosage.

### X-ray absorption near-edge structure (XANES) measurement

XANES studies were performed at the bending magnet beamline, 12-BM-B, at the Advanced Photon Source, Argonne National Laboratory. The linear polarized X-rays after the Si (111) monochromator with resolution δE/E = 1.4 × 10^–4^ has a total flux of 2 × 10^11^ photons/s. The absorption spectra were collected by the fluorescence mode with samples mounted in a custom-designed X-ray cell allowing in-situ electrochemical control of gating bias during ILG. A 13-element Ge drift detector (Canberra) was used to measure the fluorescence yield. Glancing incidence geometry (e.g., >4–5 times of the substrate critical angle) was adopted to cover the signal contributed by the whole SrRuO_3_ film, as well as to minimize the elastic scattering background. A Ru metal foil was used as an online check of the monochromator energy calibration. The originally collected XANES spectra were normalized by fitting the pre-edge to zero and the post-edge to 1 using Ifeffit performed by the software Athena.

### XAS and XMCD measurements

Soft X-ray absorption spectra (XAS) and corresponding X-ray magnetic circular dichroism (XMCD) measurements at Ru M-edges were measured at beamline 4.0.2 of Advanced Light Source with total electron yield (TEY) mode. The measurements were done at 10 K under high vacuum (around 10^−8^ Torr), and the incident circularly polarized (90%) X-ray was perpendicular to the film surface, with a magnetic field of 4T applied along the beam incident direction. The XAS spectra were deduced from the average of positive (*μ*^+^) and negative (*μ*^−^) magnetic field contributions, and the XMCD spectra were taken as the difference between two magnetic field contributions.

### First-principles calculations

First-principles density-functional theory (DFT)^41,42^ calculations with the generalized-gradient approximation (GGA)^43^, local spin density approximation (LSDA) and the projector-augmented wave (PAW) method^44^ were performed using the Vienna Ab-initio Simulations Package (VASP) (http://www.vasp.at/). The calculations were performed using a plane-wave energy cutoff of 400 eV, and a Gamma mesh of 4 × 4 × 4 k-points was used for the pristine orthorhombic SrRuO_3_ and protonated superstructure. The lattice constants were fixed to the experimental results during the calculations for the pristine orthorhombic SrRuO_3_, while in the calculations for protonated superstructures, the in-plane lattice constant was fixed to 3.905 Å (lattice constant of SrTiO_3_), and the out-of-plane lattice constant was allowed to relax. All structural relaxations were computed using GGA and the magnetic energy calculations were carried out using LSDA. The atomic positions were fully optimized in all the magnetic orders until the change of the total energy is smaller than 10^−5^ eV. To obtain the atomic crystalline structure of protonated phases, we have considered the cases with the proton bonded with the epical oxygen ions, equatorial oxygen ions or both. When all protons are bonded with the epical oxygen ions, the oxygen octahedra will tilt around the *a* (or *b*) axis due to the attraction between proton and oxygen ion, and the total system energy is highest (due to strong lattice distortion). When all protons are bonded with equatorial oxygen ions, the octahedra will rotate around the *c* axis, and the total energy reaches the lowest state with the two adjacent octahedra tilted along opposite directions. We also calculated the case with protons bonded with both epical and equatorial oxygen ions, in which the total energy is between the above two cases.

The magnetic order was determined using Stoner theory^45^, according to which the paramagnetic structure was not stable and turned into ferromagnetic structure if the criterion *IN* > 1 was met, where I is the Stoner factor and N is the DOS per spin f.u. at the Fermi level in nonmagnetic structure. The Stoner parameter was calculated from the Landau free energy, which can be expressed as, ${\mathrm{E}}\left( {\mathrm{M}} \right) = {\mathrm{E}}\left( 0 \right) + \left( {\frac{{{\mathrm{a}}_2}}{2}} \right){\mathrm{M}}^2 + \frac{{{\mathrm{a}}_4}}{4}{\mathrm{M}}^4 + \ldots$, where M is the magnetic moment per formula unit. The coefficient a_2_ is given by ${\mathrm{a}}_2 = \frac{{\partial ^2{\mathrm{E}}}}{{\partial {\mathrm{M}}^2}}|_{{\mathrm{M}} = 0} = {\upchi}^{ - 1} = \frac{1}{2}\left( {\frac{1}{{{\mathrm{N}}\left( {{\mathrm{E}}_{\mathrm{F}}} \right)}} - {\mathrm{I}}} \right)$. By fixed spin moment calculations, we can calculate the energy at different magnetic moment, and derive a_2_ and ${\mathrm{IN}}\left( {{\mathrm{E}}_{\mathrm{F}}} \right)$ using polynomial fitting.

## Supplementary information

Supplementary Information

## Data Availability

All data supporting the findings of this study are available from the corresponding author on request.
