# Supplementary material for: Reversible manipulation of the magnetic state in SrRuO3 through electric-field controlled proton evolution
Source: Nat Commun. 2020 Jan 10;11:184. doi: 10.1038/s41467-019-13999-1 (PMC6954193; doi:10.1038/s41467-019-13999-1)
Supplement: Supplementary file 1 — Supplementary Information [file 41467_2019_13999_MOESM1_ESM.pdf]

Supplementary Information

Reversible manipulation of the magnetic state in $SrRuO_3$ through electric-field controlled proton evolution

Zhuolu Li, Shengchun Shen, Zijun Tian, Kyle Hwangbo, Meng Wang, Yujia Wang, F. Michael Bartram, Liqun He, Yingjie Lyu, Yongqi Dong, Gang Wan, Haobo Li, Nianpeng Lu, Jiadong Zang, Hua Zhou, Elke Arenholz, Qing He, Luyi Yang, Weidong Luo, and Pu Yu

Supplementary Note 1

Hydrogen intercalation into the $SrRuO_3$ films during the ILG. Both electrostatic charge accumulation[1] and oxygen ion evolution[2] have been reported in the previous studies of ILG on $SrRuO_3$ thin films. While in our case, the observations of both structural transformations and reduction of Ru valence state in bulk strongly suggest that the effect is not dominated by the surface electrostatic charge modulation. To trace whether the oxygen evolution is involved during ILG, we performed SIMS measurements on $SrRuO_3$ films dwelled in pure $^{18}O_2$ atmosphere during the ILG, where any oxygen evolution occurred during the ILG would result in the accumulation of ^{18}O within the film. However, the SIMS measurements reveal that the ^{18}O depth profile signals are almost identical and negligible for both pristine and gated $SrRuO_3$ films (**Supplementary Figure 3b**). Therefore, this result indicates the oxygen evolution is not essential during ILG in our case. Thus, we can conclude that the hydrogen intercalation into $SrRuO_3$ films during ILG operates as the dominant mechanism for the observed crystalline structural phase transformation and the reduction of Ru valence state across the whole film. With this, we can assign the chemical formula for the new protonated phase as H_xSrRuO_3. In order to trace the origin of the hydrogen in the H_xSrRuO_3, we doped the employed ionic liquid with heavy water (D_2O). Consequently, a significant deuterium (D) signal was observed in the gated $SrRuO_3$ film (**Supplementary Figure 3a**), which provides solid evidence to attribute the origin of hydrogen in the gated $SrRuO_3$ films to the water residual (typically around several hundred ppm) inside the ionic liquid[3, 4].

Supplementary Figures

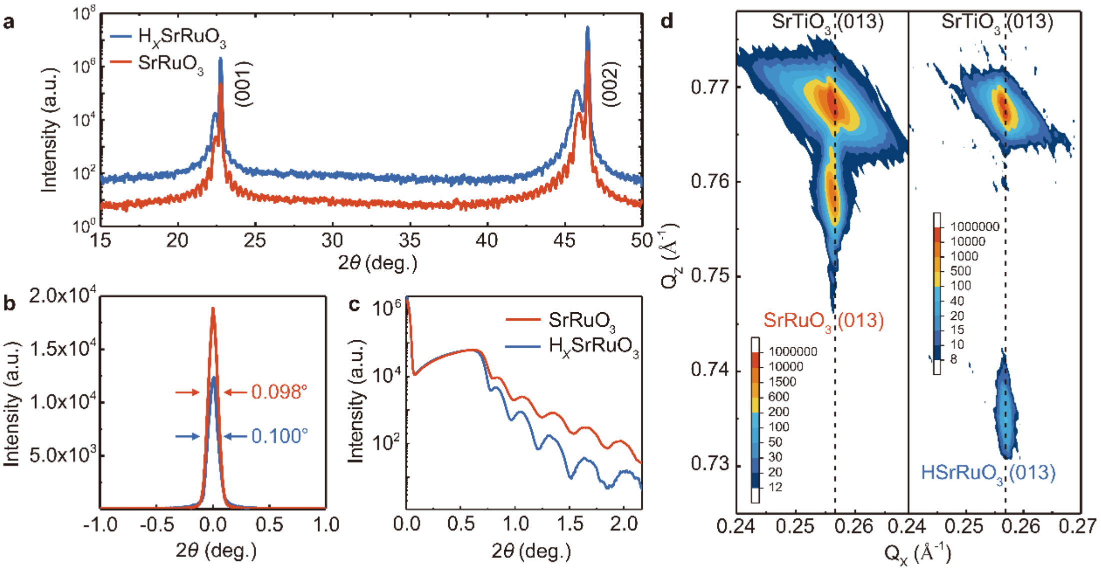

Supplementary Figure 1. Comparison of the crystalline structures between pristine and protonated $SrRuO_3$ films. **a**, XRD *θ-2θ* scans of pristine (red, 26 nm) and gated (blue) $SrRuO_3$ films, respectively. **b**, Rocking curves around (002) peak of both two phases. The full width at half maximum (FWHM) of pristine and gated films are almost identical. **c**, X-ray reflectivity curves of these two phases. The protonated samples used for the ex-situ XRD measurements shown in (**a**, **b**, **c**) were obtained with ILG at V_G = 3.5 V. **d**, Reciprocal space mapping (RSM) results around (013) peaks of STO substrates for both pristine and in-situ protonated (at V_G = 3.5 V) films.

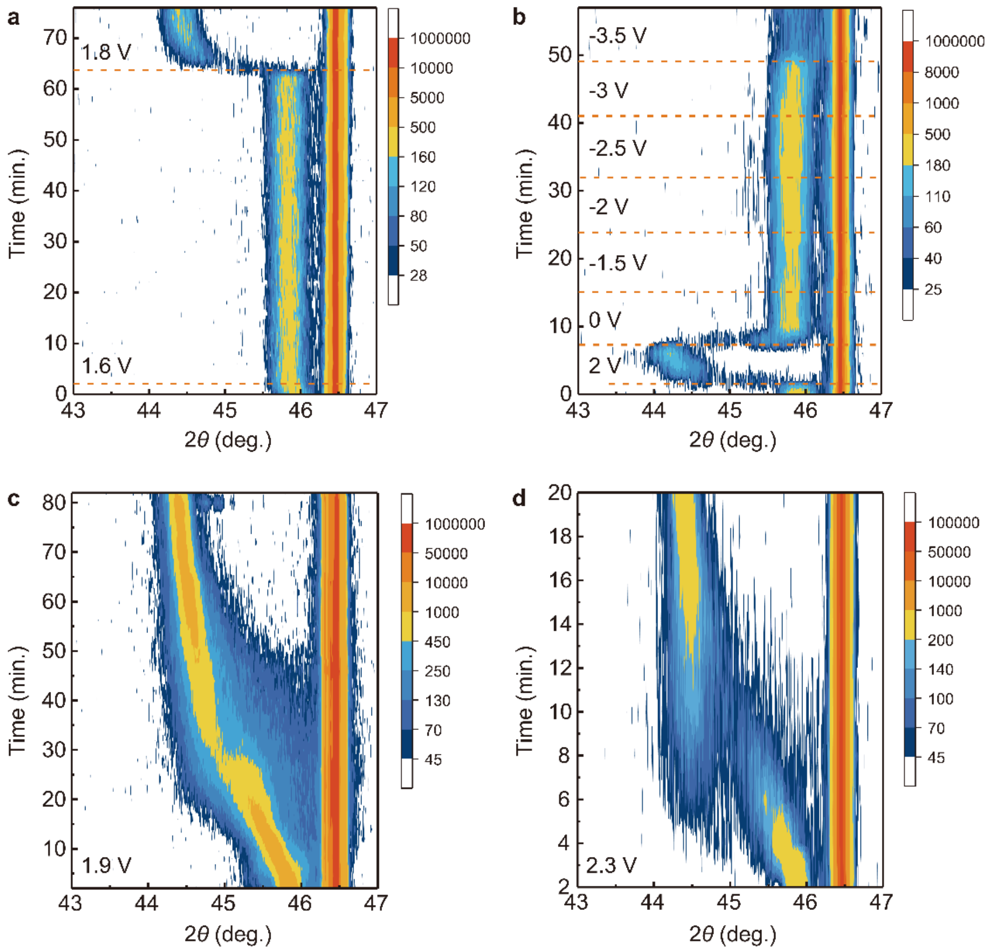

Supplementary Figure 2. In-situ XRD measurements during the ILG induced structural transformation. **a**, Gating duration dependent In-situ θ-2θ scans around (002) peak of films as fixed V_G of 1.6 and 1.8 V. **b**, In-situ XRD θ-2θ scans around (002) peak in a wide range of V_G from +2 V to -3.5 V. The protonated phase quickly returns back to a lightly protonated phase under the gating voltage of 0 V and then remains stable with the gating voltage up to -3 V, above which the film will be damaged by the ILG process. **c, d,** In-situ XRD θ-2θ scans around the $SrRuO_3$ (002) peak at (**c**) 1.9 V and (**d**) 2.3 V on the films with the same thickness of 44 nm.

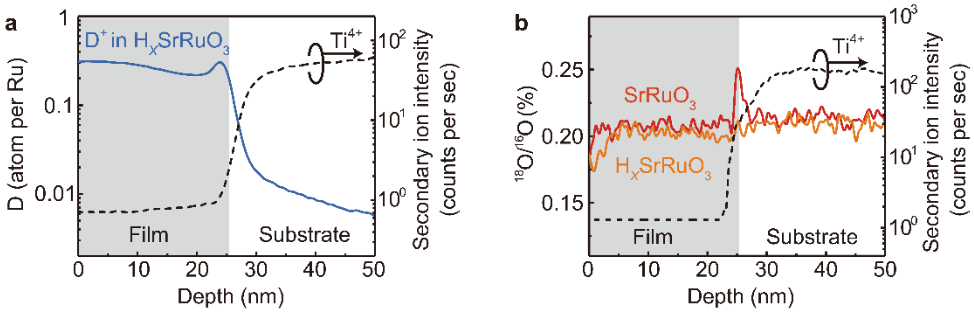

Supplementary Figure 3. Extended compositional analysis of the gated $SrRuO_3$ films. **a**, Depth profiles of the deuterium ion (D^+) in gated (V_G of 3.5 V) $SrRuO_3$ film with the heavy water doped IL. **b**, Depth profile of $^{18}O/^{16}O$ ratio in pristine $SrRuO_3$ film and the gated film relaxed in ^{18}O atmosphere. In both (**a**) and (**b**), the Ti^{4+}signal was used to define the interface position.

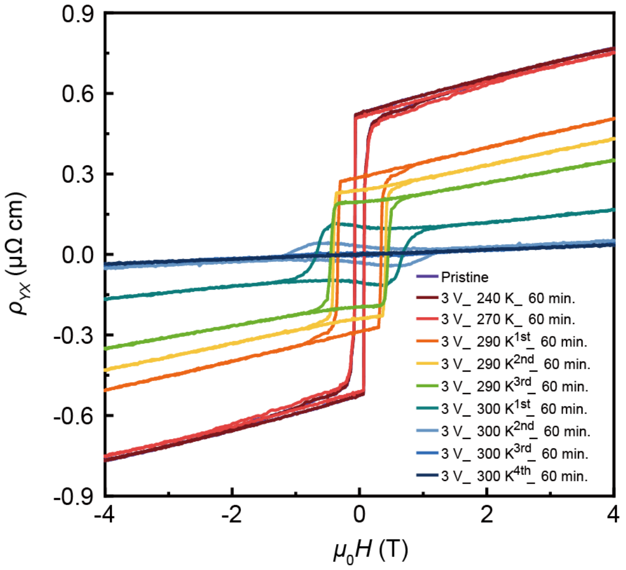

Supplementary Figure 4. Direct comparison of Hall resistivity measured at different gating conditions for a 70-nm-thick film. Gating temperature and duration dependent Hall signals measured at 50 K. The gate sequence is shown in the figure legend (from the top to bottom), with the gating temperature and dwell time changed accordingly during ILG. During this measurement, the AHE signals change only slightly when ILG performed at low temperatures (240 K and 270 K), while it is significantly suppressed when increasing the gating temperature up to 290 K. Since all temperatures are well above the glass transition temperature (~220 K) of ionic liquid used, these results can clearly exclude the electrostatic modulation scenario, and the temperature dependence could be attributed to the intrinsic proton diffusion process through its thermal activation. We repeated the same measurement at 290 K twice, and as consequence the AHE signal was gradually suppressed, matching nicely with the time dependent scenario. Afterward, we increased the gating temperature further to 300 K, and observed that the AHE was dramatically suppressed and eventually disappeared at the fourth measurements.

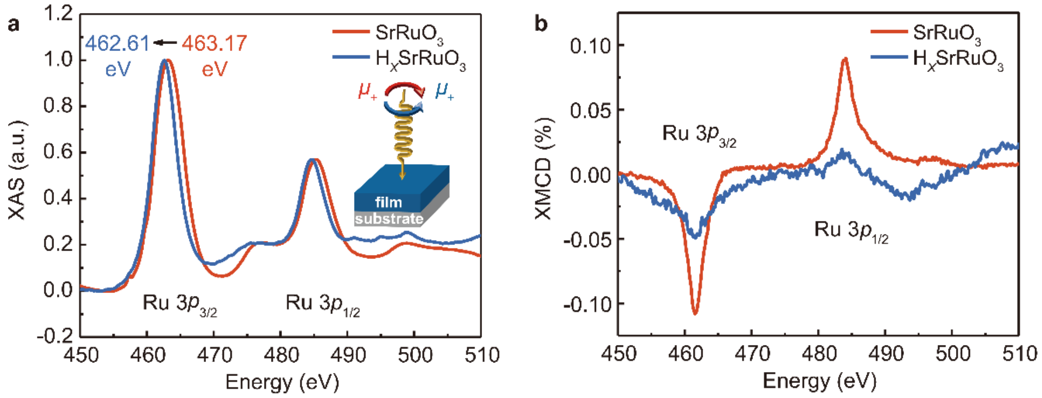

Supplementary Figure 5. | X-ray absorption spectra for pristine and protonated $SrRuO_3$ films. **a**, XAS spectra of pristine (red) and ex-situ gated (blue) $SrRuO_3$ films. The shift of peak positions for Ru M_3 and M_2 peaks to the lower energy indicates the reduced Ru valence state in the gated sample, which is consistent with our scenario of electron doping with protonation. The inset shows the experimental configuration, in which the incident μ^+ (right circularly) and μ^- (left circularly) polarized X-rays are perpendicular to the film surface. **b**, Comparison of x-ray magnetic circular dichroism (XMCD) results for both two phases. An obvious decrease in the XMCD intensity suggests a suppression of ferromagnetic contribution from Ru element. We note that the soft x-ray absorption technique is a surface technique, which probes mainly the top 5 to 10 nm of the films. While in our gated sample, although the majority portion of proton is released from the film along with the structural relaxation, there is still large amount of proton concentration at the top surface, which can nicely explain the suppressed XMCD signal as the consequence of the reduced magnetism in the protonated $SrRuO_3$.

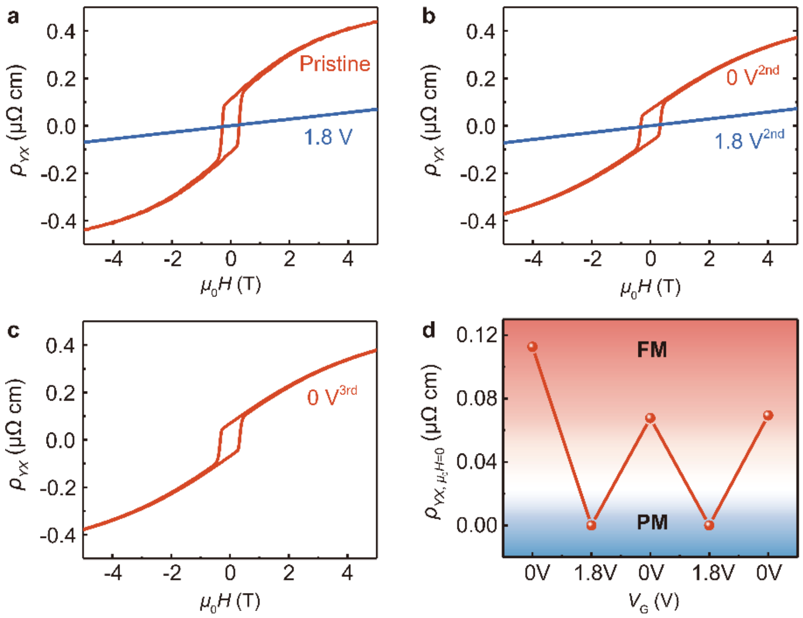

Supplementary Figure 6. Reversible control of magnetism in $SrRuO_3$ during the ILG. **a-c**, Magnetic field dependent Hall resistivity at 2 K, as the gating voltage V_G cycled between 0 V and 1.8 V. **d**, The reversible ferromagnetic phase transition along with cycled V_G.

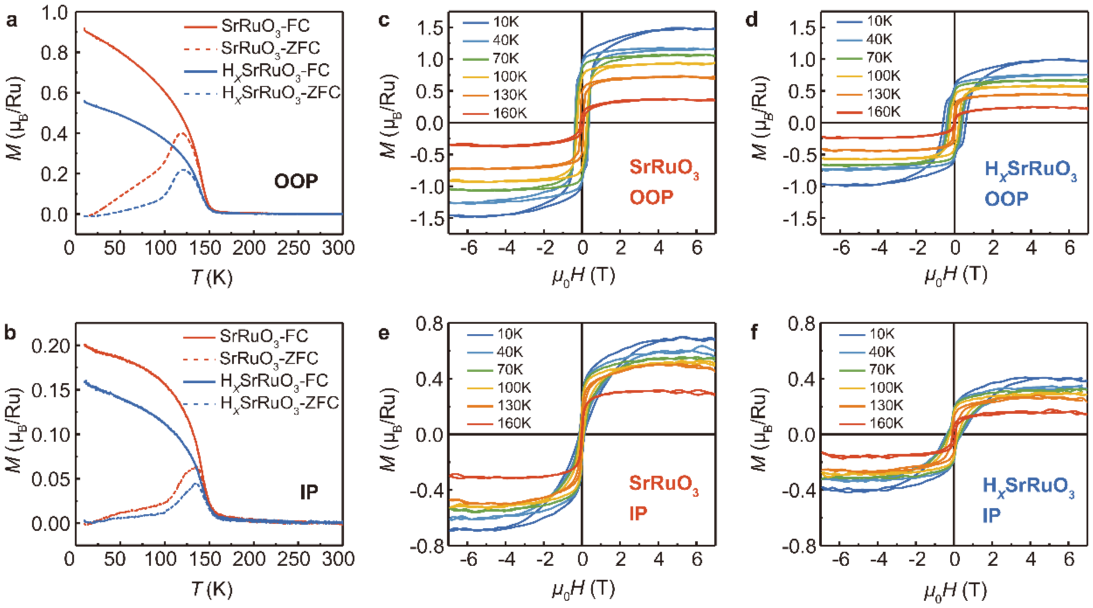

Supplementary Figure 7. Macroscopic magnetic measurements of pristine and post-gated $SrRuO_3$ films. **a-f**, Temperature dependent (**a**) out-of-plane (OOP) and (**b**) in-plane (IP) magnetization with field cooling and zero field cooling processes for both pristine and gated samples. (**c**, **d**) Out-of-plane and (**e**, **f**) in-plane magnetic hysteresis loops at different temperatures for both (**c**, **e**) pristine and (**d**, **f**) post-gated SRO films. In this measurement, the gated samples were obtained with ex-situ ILG at gating voltage of 3.5 V. The results show that a lightly reduced magnetism in the ex-situ gated sample due to the residual proton concentration within the film.

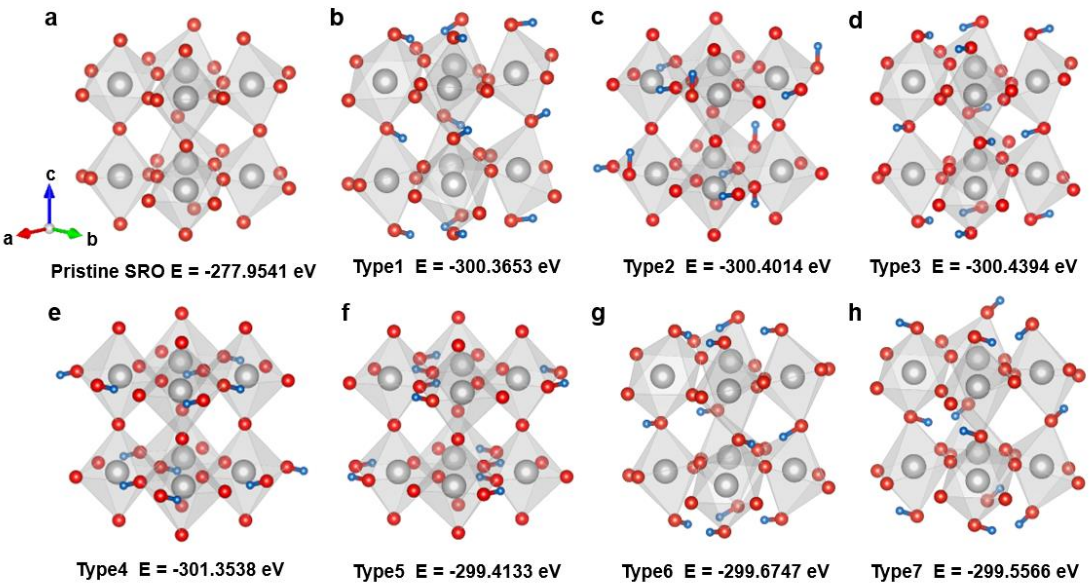

Supplementary Figure 8. Calculated representative crystalline structures of $HSrRuO_3$. The gray balls represent the ruthenium ions and the protons (blue balls) are bonded to oxygen atoms (red balls). The Sr ions are omitted in this figure. We have considered all cases with the proton bonded with the epical oxygen ions, equatorial oxygen ions or both. When all protons are bonded with the epical oxygen ions, the oxygen octahedra will tilt around the a (or b) axis due to the attraction between proton and oxygen ion, and the total system energy is highest due to strong lattice distortion. When all protons are bonded with equatorial oxygen ions, the octahedra will rotate around the c axis, and the total energy reaches the lowest state in the case the two adjacent octahedra are tilted along opposite directions. We also calculated the case with protons bonded with both epical and equatorial oxygen ions, in which the total energy is between the above two cases. The lowest energy configuration is Type4 (**Fig. S8e**), in which the protons are bonded with the equatorial oxygen and the lattice shows the least distortion.

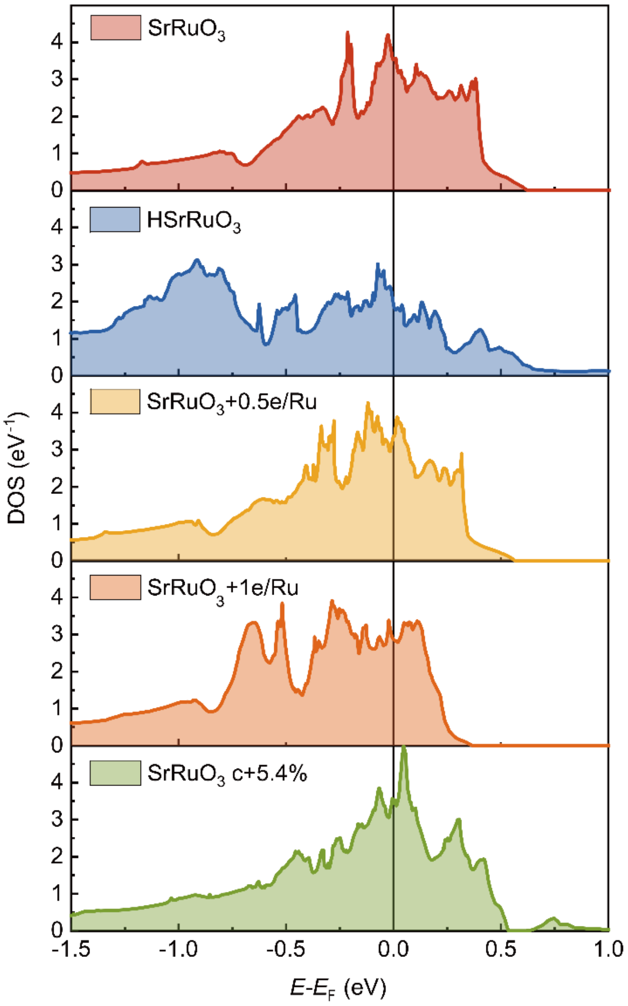

Supplementary Figure 9. Calculated non-spin-polarized density of states. The calculations were carried out in the cases of pristine $SrRuO_3$, protonated $HSrRuO_3$, $SrRuO_3$ with additional 0.5 electron per Ru, $SrRuO_3$ with additional one electron per Ru and $SrRuO_3$ with only 5.4% lattice expansion, respectively.

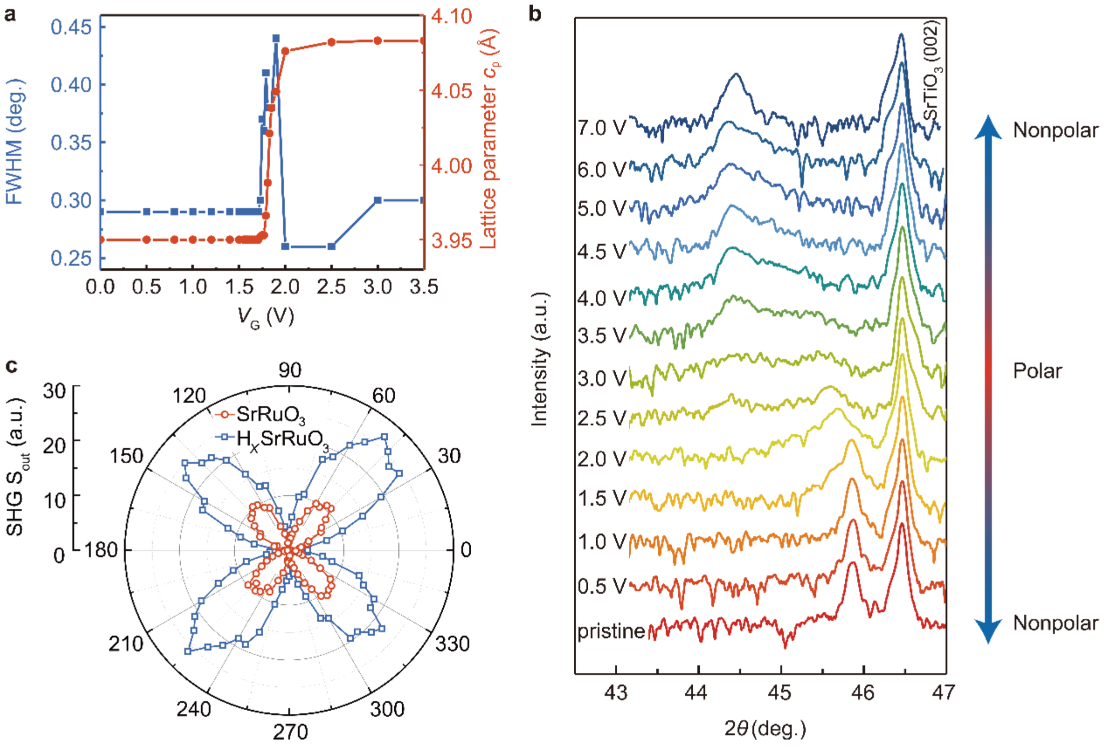

Supplementary Figure 10. Emergence of a polar state during the ILG induced structural transformations. **a**, Correlation between the full width at half maximum (FWHM) of XRD *θ*-*2θ* (002) diffraction peak of $SrRuO_3$ film (presented in **Fig. 1,** 28 nm) and the *c*-axis lattice constants as a function of V_G. **b**, Detailed gating voltage dependent XRD *θ*-*2θ* scans for a 90-nm $SrRuO_3$ thick film, in which a gradual structural-transformation is suggested based on the evolution of diffraction peaks. This result can be attributed to the formation of depth dependent proton concentration during the ILG, which is more pronounced around the critical gating voltage. **c**, Comparison of the *s*-polarized SHG intensity profiles for both pristine $SrRuO_3$ and gated H_xSrRuO_3 films. The enhanced SHG signal in the gated sample suggests the induced crystalline inversion symmetry with the proton concentration gradient.

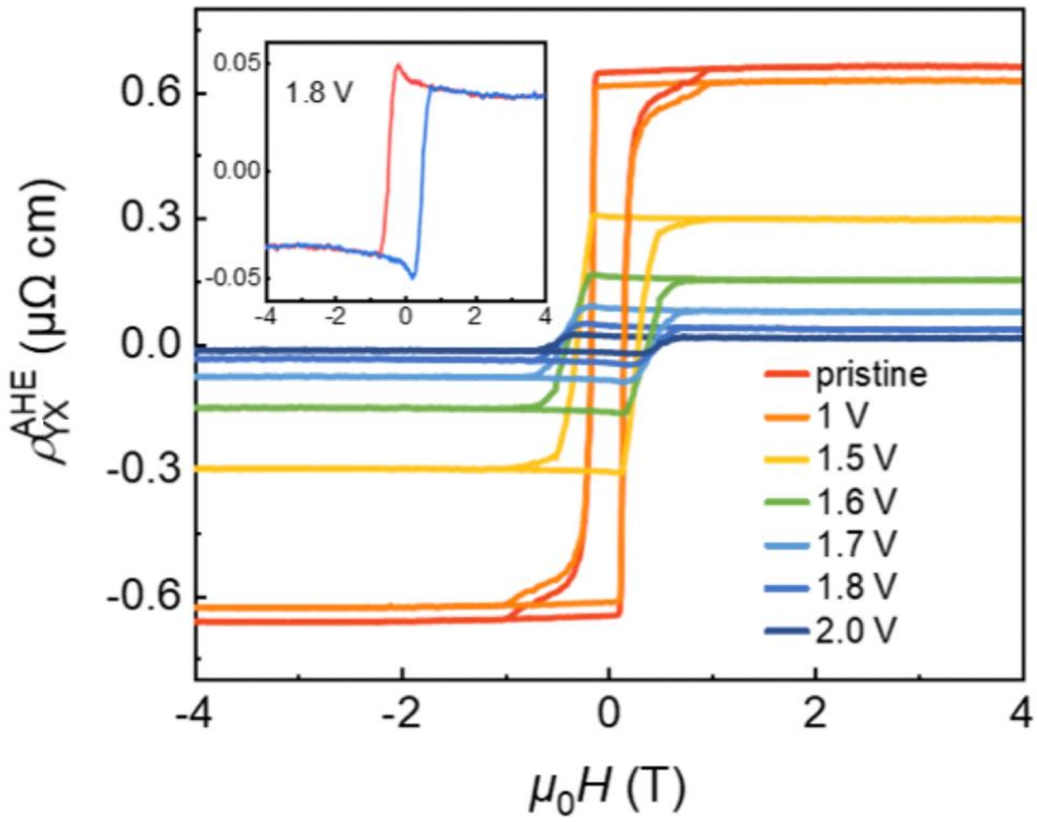

Supplementary Figure 11. The magnetic field dependent anomalous Hall resistivity measured at 80 K with different gate voltage. The results were measured with a similar sample with thickness of 28 nm as the one obtained MOKE data (shown in **Fig. 2**), and the conventional Hall signal was subtracted through a linear fitting for the high-field data.

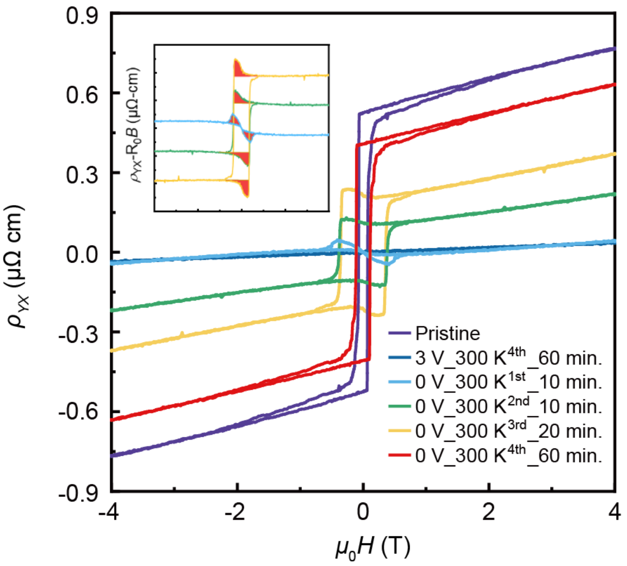

Supplementary Figure 12. Evolution of AHE signals from a fully protonated sample when the voltage was turned off. The measurements were carried out at 50 K with the sample (~70 nm) after **Fig. S4**. The inset shows the emergence of topological Hall effect (estimated topological Hall resistivity is marked by red) across the magnetic transition.

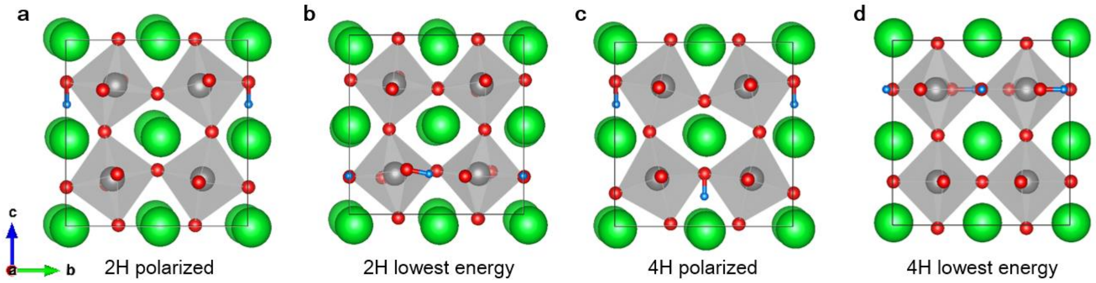

Supplementary Figure 13. Representative polar and nonpolar crystalline structures for two protonated $SrRuO_3$ phases. The green, gray, red and blue balls represent the strontium ions, ruthenium ions, oxide ions and protons, respectively. To mimic the protonation phases, we built up the supercell with 8 formula units of $SrRuO_3$ and then arbitrarily introduced 2 and 4 hydrogen ions into the lattice, leading to $H_{0.25}SrRuO_3$ and $H_{0.5}SrRuO_3$ as the intermediate protonated phases. For both cases, the lattice relaxation would lead to a nonpolar crystalline structure (as shown in **b** and **d**) as ground state, with all ferroelectric phases tested return eventually back to the nonpolar phase after the structural optimization. For instance, the calculated polar state with the deviation of hydrogen ions between the Sr plane and O plane for about 0.24 Å (~ 0.2 Å) in 2H (4H) structure (**a and c**) forms only metastable state with the total energy of 68 meV (255 meV) higher than its corresponding non-polar ground state.

Supplementary Table 1. Summary of the samples used in the manuscript.

Result	Experiment	Thickness (nm)	Substrate
1a, S10a	In-situ XRD	28	$SrTiO_3$
1b	In-situ XRD	33	$SrTiO_3$
1c	Ex-situ SIMS	31	$SrTiO_3$
1d	In-situ XNAES	28	LSAT
2a, 2b, 2c,2d	In-situ transport	32	$SrTiO_3$
2e, 2f, S11	In-situ MOKE	28	$SrTiO_3$
4a	Ex-situ SIMS	30	$SrTiO_3$
4b, S10c	Ex-situ SHG	28	$SrTiO_3$
4c, 4d	In-situ transport	32	$SrTiO_3$
S1a, S1b, S1c	Ex-situ XRD	26	$SrTiO_3$
S1d	In-situ XRD	26	$SrTiO_3$
S2a	In-situ XRD	28	$SrTiO_3$
S2b	In-situ XRD	28	$SrTiO_3$
S2c	In-situ XRD	33	$SrTiO_3$
S2d	In-situ XRD	33	$SrTiO_3$
S3a	Ex-situ SIMS	25	$SrTiO_3$
S3b	Ex-situ SIMS	25	$SrTiO_3$
S4, S12	In-situ transport	70	$SrTiO_3$
S5	Ex-situ XAS and XMCD	33	$SrTiO_3$
S6	In-situ transport	32	$SrTiO_3$
S7	Ex-situ MPMS	25	$SrTiO_3$
S10b	In-situ XRD	90	$SrTiO_3$

Supplementary References

1. Shimizu, S. et al. Gate tuning of anomalous Hall effect in ferromagnetic metal $SrRuO_3$. *Appl. Phys. Lett.* **105**, 163509 (2014).
2. Yi, H.T., Gao, B., Xie, W., Cheong, S.-W. & Podzorov, V. Tuning the metal-insulator crossover and magnetism in $SrRuO_3$ by ionic gating. *Sci. Rep.* **4**, 6604 (2014).
3. Lu, N. et al. Electric-field control of tri-state phase transformation with a selective dual-ion switch. *Nature* **546**, 124-128 (2017).
4. Wang, M. et al. Electric-Field-Controlled Phase Transformation in WO_3 Thin Films through Hydrogen Evolution. *Adv. Mater.* **29**, 1703628 (2017).
